# Comparison of the Ability to Predict Mortality between the Injury Severity Score and the New Injury Severity Score: A Meta-Analysis

**DOI:** 10.3390/ijerph13080825

**Published:** 2016-08-16

**Authors:** Qiangyu Deng, Bihan Tang, Chen Xue, Yuan Liu, Xu Liu, Yipeng Lv, Lulu Zhang

**Affiliations:** Department of Military Health Management, College of Health Service, Second Military Medical University, 800 Xiangyin Rd., Shanghai 200433, China; smmudqy@163.com (Q.D.); mangotangbihan@126.com (B.T.); xue1990chen@163.com (C.X.); yawnlau@126.com (Y.L.); xuliuaqua@126.com (X.L.); happylvyipeng@163.com (Y.L.)

**Keywords:** Injury Severity Score, New Injury Severity Score, mortality, meta-analysis

## Abstract

*Background:* Description of the anatomical severity of injuries in trauma patients is important. While the Injury Severity Score has been regarded as the “gold standard” since its creation, several studies have indicated that the New Injury Severity Score is better. Therefore, we aimed to systematically evaluate and compare the accuracy of the Injury Severity Score and the New Injury Severity Score in predicting mortality. *Methods:* Two researchers independently searched the PubMed, Embase, and Web of Science databases and included studies from which the exact number of true-positive, false-positive, false-negative, and true-negative results could be extracted. Quality was assessed using the Quality Assessment of Diagnostic Accuracy Studies checklist criteria. The meta-analysis was performed using Meta-DiSc. Meta-regression, subgroup analyses, and sensitivity analyses were conducted to determine the source(s) of heterogeneity and factor(s) affecting the accuracy of the New Injury Severity Score and the Injury Severity Score in predicting mortality. *Results:* The heterogeneity of the 11 relevant studies (total *n* = 11,866) was high (I^2^ > 80%). The meta-analysis using a random-effects model resulted in sensitivity of 0.64, specificity of 0.93, positive likelihood ratio of 5.11, negative likelihood ratio of 0.27, diagnostic odds ratio of 27.75, and area under the summary receiver operator characteristic curve of 0.9009 for the Injury Severity Score; and sensitivity of 0.71, specificity of 0.87, positive likelihood ratio of 5.22, negative likelihood ratio of 0.20, diagnostic odds ratio of 24.74, and area under the summary receiver operating characteristic curve of 0.9095 for the New Injury Severity Score. *Conclusion:* The New Injury Severity Score and the Injury Severity Score have similar abilities in predicting mortality. Further research is required to determine the appropriate use of the Injury Severity Score or the New Injury Severity Score based on specific patient condition and trauma type.

## 1. Introduction

With the recent increase in traffic, the degree of trauma has become increasingly serious. In addition, disasters such as earthquakes, tsunamis, and typhoons occur frequently. Trauma has become the leading cause of morbidity and mortality among individuals aged <40 years and is the third main cause for death worldwide [1]. Meanwhile, there are approximately 5 million deaths due to injuries annually worldwide [2,3].

Following an injury, timely description of the anatomical severity of injuries in trauma patients is important. The Injury Severity Score (ISS) has been considered the “gold standard” [4] indicator for anatomical injury severity since it was first introduced in 1974 [5]. The ISS is the sum of the squares of the single highest Abbreviated Injury Scale score for each of the three most severely injured body regions. However, it only considers one injury per body region. Therefore, in 1997, Osler et al. [6] introduced a modification to the ISS to improve its accuracy and named it the New Severity Injury Score (NISS). The NISS is the sum of the squares of the three highest Abbreviated Injury Scale scores for each patient, regardless of body region.

Various subsequent studies in different countries have used different methodologies to compare the predictive capacities of the ISS and NISS for mortality. A number of these studies found that the NISS is superior to the ISS [7,8,9,10,11,12,13], particularly for predicting mortality in blunt trauma patients [14]. However, because the two tools share similar accuracy and calibration, it has recently been suggested that the NISS should not replace the ISS [15,16,17,18,19,20]. Moreover, the ISS reportedly has a superior ability to predict both intensive care unit admissions and length of hospital stay compared to the NISS [21].

Despite the lack of consistent results, a meta-analysis has not previously been performed, due to the heterogeneity among studies, as identified in a previous systematic review [14]. The present diagnostic meta-analysis aimed to determine if the NISS is better than the ISS for predicting mortality.

## 2. Materials and Methods

### 2.1. Search Strategy and Study Selection

Studies of the predictive ability of the NISS and ISS for mortality were included in this meta-analysis, irrespective of publication status. Two investigators (Qiangyu Deng and Bihan Tang) conducted a systematic search for literature using the electronic databases PubMed, Embase, and Web of Science (all studies until 21 July 2015). The following search terms were used in PubMed: “((New Injury Severity Score OR NISS) OR (Injury Severity Score OR ISS)) AND (((sensitivity OR specificity) OR receiver operating characteristic curve) OR ROC)”. The following search terms were used in Embase and Web of Science: New Injury Severity Score, or NISS, or Injury Severity Score, or ISS; and sensitivity, or specificity, or receiver operating characteristic curve, or ROC. Additionally, manual searches of references cited in all relevant original and review articles were conducted. If the full text was not available in the databases, we attempted to obtain the information from the authors by email.

### 2.2. Inclusion and Exclusion Criteria

For studies to be considered eligible for inclusion in the meta-analysis, they should have assessed the performance of the ISS or NISS in predicting patient mortality, and the exact number of true-positive, false-positive, false-negative, and true-negative test results could be either directly or indirectly extracted. The exclusion criteria were as follows: did not assess the performance of ISS or NISS for predicting patient mortality; were not published in English; and were conference abstracts or letters to editors. If more than one article reported data from the same population, then the most recent and complete article was included in the meta-analysis. The two investigators who conducted the literature search also independently performed the study selection and decisions regarding inclusion criteria (Qiangyu Deng and Bihan Tang).

### 2.3. Data Extraction and Quality Assessment

The following data were independently extracted from each eligible study by two investigators (Qiangyu Deng and Bihan Tang): the first author’s surname, year of publication, study location, proportion of male subjects, age, study design, number of cases, sensitivity, specificity, area under the receiver operator characteristic (ROC) curve (AUC), and cut-off value.

Quality assessments were independently conducted by two investigators (Qiangyu Deng and Bihan Tang) using a 14-item instrument recommended by the Quality Assessment of Diagnostic Accuracy Studies (QUADAS) [22] checklist criteria. This instrument is widely used as a quality assessment tool for systematic reviews of diagnostic studies. The meta-analysis included studies with positive answers for >7 questions on the tool because studies with positive answers for ≥8 questions are considered high-quality studies. The quality assessment of the original articles was reexamined and independently adjudicated by an additional investigator (Lulu Zhang).

### 2.4. Statistical Analysis

Heterogeneity was evaluated using the I^2^ test, and thresholds of 25%, 50%, and 75% indicated low, moderate, and high heterogeneity, respectively. We used Spearman coefficient analysis to test the threshold effect, which reflects the correlation between sensitivity and 1-specificity. The cumulative parameters and 95% confidence interval (CI) of the sensitivity, specificity, positive likelihood ratio (PLR), negative likelihood ratio (NLR), diagnostic odds ratio (DOR), and AUC were then calculated and pooled.

A meta-regression analysis was conducted to test the effect of the number of patients, mortality, cut-off values for the NISS and ISS, and study quality on the diagnostic power of NISS and ISS for mortality. After excluding specific studies, sensitivity analyses were performed to explore if these exclusions would significantly affect the results.

Subgroup analyses were performed for groups based on mortality, number of patients, cut-off value, and country. Mortality reflected the injury severity; the group of serious injuries included mortality ≥0.1, which indicates that ≥10% of the patients in these studies died, and the group of slight injuries included mortality <0.1. A small sample was defined in the inclusion as <100 patients. The cut-off values were grouped using the definitions by Osler et al. [6]. Countries were grouped according to economic characteristics, namely developed and developing countries.

All statistical analyses were performed using Meta-DiSc 1.4 (Hospital Ramon y Cajal and Universidad Complutense de Madrid, Madrid, Spain) [23]. A *p* value < 0.05 was considered statistically significant.

## 3. Results

### 3.1. Search Results and Characteristics of Studies

**Figure 1:**
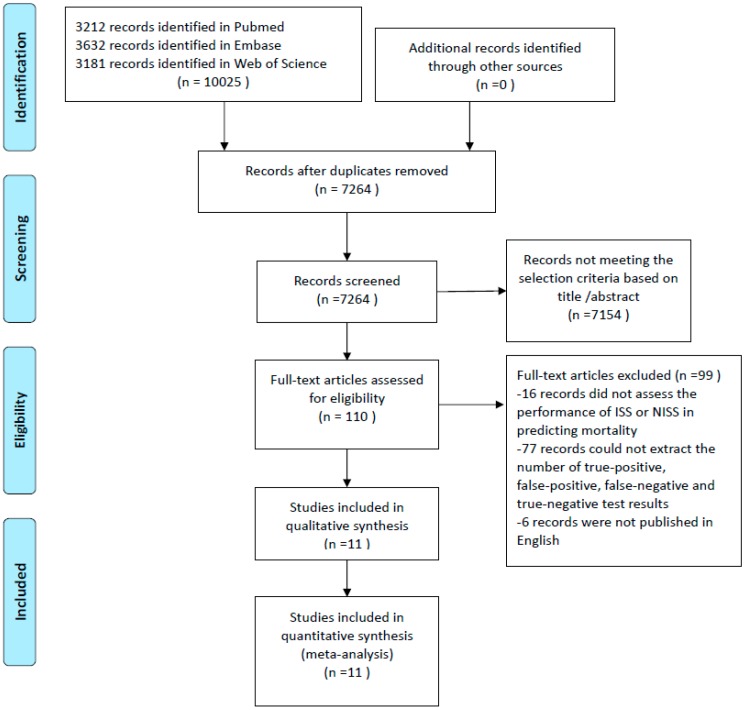
Flow chart showing study selection for the meta-analysis regarding the ability of the Injury Severity Score and New Injury Severity Score to predict mortality.

The search strategy resulted in 7264 studies after removing the duplicates using EndNote (Figure 1). After reading the title and abstract and excluding studies that did not use the ISS or the NISS as a prognostic method to predict mortality, the full text of 110 studies were evaluated, and 16 studies were excluded because they did not assess the performance of the ISS or NISS in predicting mortality. An additional 77 studies were excluded because the number of true-positive, false-positive, false-negative, and true-negative test results could not be extracted, and six records were excluded because they were not published in English. Finally, 11 full-text articles assessing the performance of the ISS or NISS for predicting mortality were included in the meta-analysis (Table 1).

The studies originated from seven countries, including America, Brazil, Germany, Iran, and China (Taiwan). The number of patients ranged from 41 to 7208 (total *n* = 11,886), with all studies including adults, except for one study that included children aged <14 years [18]. Because one study [24] included two groups of patients, and provided results for each group rather than an overall result, we considered the two groups separately in the analysis. More than 50% of the sample in most of the studies comprised men, while one study [25] included only pregnant women. Five of the 11 studies discussed both the ISS and NISS, six studies discussed only the ISS, and none of the studies discussed only the NISS. Most of the patients were based in hospitals, while one study included patients from The Trauma Registry of the German Society for Trauma Surgery (TR-DGU) [26] and one included patients from a level I trauma center [13]. The cut-off value and mortality were also different between studies, and the cut-off value indicates a threshold for predicting mortality. All true-positive, false-positive, false-negative, and true-negative test results were indirectly extracted from the sensitivity, specificity, mortality values and the number of patients. The results of the quality assessment using the QUADAS checklist criteria are also shown in Table 1. The median number of positive answers on the checklist was 9 (range, 8–11).

### 3.2. Test of the Threshold Effect and Heterogeneity

The Spearman coefficient analysis to test the threshold effect resulted in positive Spearman correlation coefficients for the sensitivity and specificity of the NISS and ISS (Table 2), indicating no threshold effect. However, the I^2^ values between studies were high (sensitivity I^2^ = 93.2%; specificity I^2^ = 99.3%; PLR I^2^ = 97.0%; NLR I^2^ = 87.5%; DOR I^2^ = 94.0%), indicating high levels of heterogeneity. The heterogeneity might have originated from the differences in the samples, cut-off values, or methods between studies, rather than a threshold effect.

### 3.3. Overall Analysis

Because of the high level of heterogeneity (I^2^ > 80%), we pooled related statistical parameters (Table 3) using a random-effects model. For the ISS, the pooled estimates were 0.64 (95% CI: 0.61–0.68) for sensitivity, 0.93 (95% CI: 0.93–0.94) for specificity, 5.11 (95% CI: 3.12–8.37) for PLR, 0.27 (95% CI: 0.19–0.40) for NLR, and 27.75 (95% CI: 9.93–77.53) for DOR. For the NISS, the pooled estimates were 0.71 (95% CI: 0.66–0.75) for sensitivity, 0.87 (95% CI: 0.86–0.88) for specificity, 5.22 (95% CI: 3.84–7.08) for PLR, 0.20 (95% CI: 0.08–0.52) for NLR, and 24.74 (95% CI: 10.19–60.07) for DOR.

**Figure 2:**
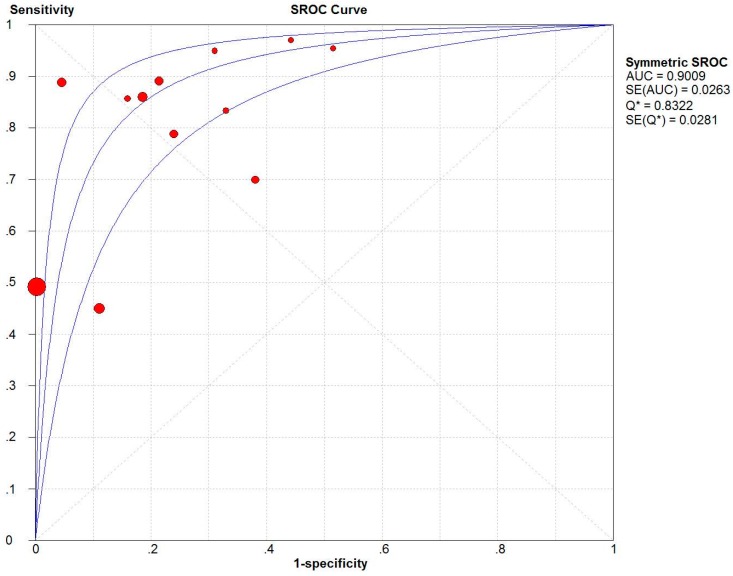
Summary receiver operating characteristics (SROC) curve of the Injury Severity Score (ISS). AUC is the area under the receiver operator characteristic (ROC) curve.

**Figure 3:**
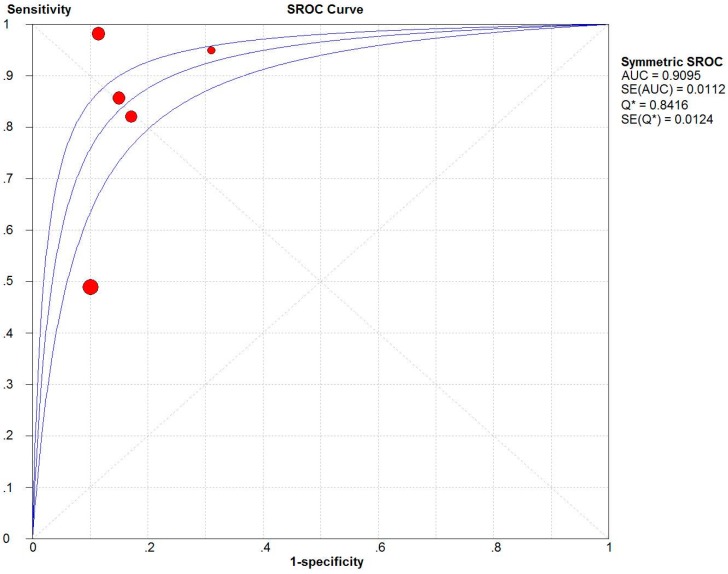
Summary receiver operating characteristics (SROC) curve of the New Injury Severity Score (NISS). AUC is the area under the receiver operator characteristic (ROC) curve.

The two summary ROC (SROC) curves [32] for the ISS (Figure 2) and NISS (Figure 3) plot the true-positive rates against the false-positive rates from each study. The AUC for the NISS (AUC = 0.9095) was similar to that for the ISS (AUC = 0.9009).

### 3.4. Meta-Regression and Sensitivity Analyses

The meta-regression analysis [33] was conducted because of the high level of heterogeneity between studies (I^2^ > 80%); the number of patients, mortality, cut-off value, and quality of studies did not affect the diagnostic accuracy (Table 4).

To determine the source of heterogeneity, sensitivity analysis was conducted (Table 5). The study by Turina et al. [24] was excluded because it was the only study including patients with war-related injuries (ballistic injuries); the study by Domingues et al. [13] was removed because the patients were from a level I trauma center in America; and the studies by Bulut et al. [18] and Schiff et al. [25] were removed because the samples included only children (<14 years old) and pregnant women, respectively. The sensitivity value did not change, and the I^2^ value for sensitivity was similar to that obtained without excluding the studies. However, after removing the study by Lefering [26] because the patients were from the TR-DGU, the sensitivity increased, and the I^2^ of sensitivity decreased.

### 3.5. Subgroup Meta-Analysis

Because of the high level of heterogeneity, we performed subgroup meta-analyses by grouping studies according to mortality, number of patients, cut-off value, and country. The changes in specificity and sensitivity values are reported in Table 6. Only five studies analyzed the power of the NISS, which was not adequate to perform a subgroup meta-analysis in detail for NISS; hence, we performed subgroup meta-analysis for ISS only.

## 4. Discussion

Some recently developed trauma score systems may even discard the ISS or NISS in favor of the worst and the second worst injury. However, the ISS is by far the most frequently used severity score, and has become a kind of gold standard for traumatologists. In most instances, ISS is calculated days to weeks after injury and is only available after appropriately trained medical personnel have calculated the score based on the injuries identified. It is a very useful epidemiologic tool that can be used to guide the design of trauma care capabilities—from the capabilities of the individual center to the trauma system itself. The NISS and ISS have been compared since the NISS was developed, and even now there is no consistent result. However, understanding the abilities of the NISS and ISS to predict mortality is also important. To the best of our knowledge, this is the first diagnostic meta-analysis to study the accuracy of the NISS and ISS.

### 4.1. ISS vs. NISS

Although the NISS was developed because the ISS reportedly did not adequately consider multiple injuries in the same body region [6], the ISS and NISS had similar accuracy in predicting mortality in the present meta-analysis. The sensitivity and PLR of the NISS were slightly higher than those of the ISS, while the specificity, NLR, and DOR of the NISS were slightly lower than those of the ISS.

Likelihood ratios [34] combine the stability of sensitivity and specificity to provide an omnibus index of test performance, which is far more useful than its constituent parts. The PLR of the NISS (5.22) and that of the ISS (5.11) are similar, with a positive NISS or ISS outcome indicating an approximately five-fold higher risk of mortality. However, we should consider that a PLR should be at least 10 in order to be useful. The lower NLR for the NISS (0.20) suggests that it had a higher accuracy than the ISS (0.27) for predicting survival.

The DOR [35] is a single indicator of test accuracy that combines the sensitivity and specificity data into a single number, ranging from zero to infinity. Higher values indicate better discriminatory performance (higher accuracy) of a test, and a DOR of 1.0 indicates that the NISS or ISS does not discriminate between survival and mortality. In our study, the lower DOR for the NISS (DOR = 24.74) indicates that the NISS has less ability than the ISS (DOR = 27.75) to distinguish between survival and mortality.

The SROC summarizes the sensitivity and specificity, and is an index that is not affected by the threshold effect [32], making it more effective than the other indices. Based on the SROC curve in the present study, the AUC of the NISS (0.9095) was similar to that of the ISS (0.9009).

All of these indices indicate that the overall accuracy of both the NISS and ISS in predicting mortality was as high as expected, and they both had medium sensitivity and high specificity. In the five studies evaluating both the ISS and NISS, four studies [17,25,28,29] concluded that the accuracy of the ISS and NISS for predicting mortality was similar, while another study [13] concluded that the NISS is superior to the ISS. Therefore, we conclude that the ISS and NISS had similar accuracy for predicting mortality, in contrast with the findings of Baker [5], who developed the ISS, and Osler et al., who developed the NISS and found better prediction of mortality with the NISS than with the ISS [6].

### 4.2. Factors Affecting the Accuracy of the ISS

Some factors affected the accuracy of ISS in distinguishing between death and survival. The accuracy of the ISS changed for different subgroups. Sensitivity and specificity values were higher with sample sizes <100 and lower for studies from developed countries. Sample sizes <100 were present in the developing countries of Croatia and Turkey, where war, terrorism, or disaster occur more frequently; therefore, the ISS system is used often and correctly, resulting in better sensitivity. However, studies with a lower sample size always show better results, because moderate results in small samples will not be published. Therefore, the publication bias here should be considered when explaining the results.

The specificity was also lower with mortality ≥0.1, suggesting that the ISS performs worse in patients with severe injuries. The sensitivity after removing the study by Turina et al. did not change, suggesting that the power of the ISS for predicting mortality was not affected by patients with war-related injuries (e.g., ballistic injuries). Moreover, the sensitivity was not affected when the study by Domingues et al. was removed, which included patients from a level-I trauma center in America, indicating that the ISS has the same power in predicting the mortality of patients from a level-I trauma center or a hospital. The inclusion of children and pregnant women also did not affect the sensitivity of the ISS. Only when the study by Lefering was removed did the sensitivity increase. The TR-DGU study [26] included patients from 88 hospitals in Germany, Austria, and Switzerland, potentially resulting in different populations, which decreased the sensitivity of the ISS.

### 4.3. Source of Heterogeneity

The high level of heterogeneity among studies was not affected by the threshold effect; however, the meta-regression analysis did not determine an obvious reason. After removing the study by Rolf et al. from the sensitivity analysis, the I^2^ of sensitivity decreased. Therefore, the heterogeneity might also have been affected by the differences in the countries and populations in Germany, Austria, and Switzerland, which supports the results of Tohira et al. [14]. The subgroup meta-analysis showed that the study quality, number of patients (sample size < 100, I^2^ = 0.0%, or I^2^ = 56.8%), and differences in the economic characteristics between countries (developed country, I^2^ = 71.6%) contributed to the heterogeneity. Therefore, larger sample sizes and higher study quality contributed more to the heterogeneity. However, publication bias should be considered when explaining the influence of sample sizes on heterogeneity. The difference in studies from developed and developing countries might also have been large; in developed countries, more studies would observe a uniform standard, and a better economy would provide better health services for patients. Therefore, there was less heterogeneity among these studies. In addition, mortality and the cut-off value had little influence on heterogeneity in the subgroup meta-analysis.

### 4.4. Limitations

This meta-analysis had certain limitations. First, we included only studies published in English, which might have excluded other good studies written in other languages, such as German or Korean; Second, only one study [2] evaluated the ISS and mortality rates in different age groups. Therefore, owing to the limited detail, we were not able to analyze certain factors—such as age, treatment, cure, medical department, ethnic group, and injury type—which might influence the accuracy of the NISS or ISS; Third, most of the studies were retrospective reviews—only one study was prospective [31]. Therefore, well-designed prospective studies are required. Fourth, only two injury severity scoring systems were included in the meta-analysis. There are other tools, including the Trauma and Injury Severity Score [14], International Classification of Diseases-based ISS [14], and Severity Characterization of Trauma [36]. However, because few studies that evaluated these tools met the standards for this meta-analysis, we excluded these tools from this review. Fifth, because of the limited data for meta-analysis, in this study, we discussed only their ability to predict mortality, and excluded age and physiology, which are two of the three determinants of trauma outcome. Despite these limitations, to our knowledge, this is the first meta-analysis focusing on the NISS and ISS for predicting mortality. Our study helps to highlight areas that would benefit from further investigation.

## 5. Conclusions

In conclusion, our study demonstrated that the NISS is similar to the ISS for predicting mortality. The sources of heterogeneity and factors affecting their accuracy for distinguishing between mortality and survival were assessed using meta-regression analysis, subgroup analysis, and sensitivity analysis. These findings are valuable for understanding if the NISS is superior to the ISS and how to compare the ISS and NISS. This research might provide directions when using the NISS and ISS for differential prediction of mortality and survival for patients. However, further research is required to determine the appropriate use of the ISS or NISS based on the specific condition of the patient and the type of trauma.

## Figures and Tables

**Table 1:** General characteristics of studies that assessed the performance of the ISS or NISS for predicting mortality.

First Author	Country	Sample Size	Mortality	Year	Age (Years)	Male (%)	Tool	Cut-Off Value	TP	FP	FN	TN	AUC	Sen (%)	Spe (%)	Quality
Chiang [2]	China (Taiwan)	955	0.0450	2012	≥18	59.8	ISS	15	37	169	6	743	0.877	85.70	81.50	11
Eftekhar [27]	Iran	7208	0.0380	2005	Mean, 32.5	76.0	ISS	44 **^a^**	135	14	139	6920	0.944	49.20	99.80	10
Lefering [26]	Germany	1206	0.1660	2009	Mean, 38.2	74.0	ISS	40	90	111	110	895	0.786	45.00	89.00	10
							NISS	49	98	101	102	905	0.804	49.00	90.00	
Bulut [18]	Turkey	749	0.0360	2006	<14	64.0	ISS	22	24	33	3	689	0.962	90.50	95.40	9
							NISS	22	27	82	0	640	0.950	100.00	88.70	
Woodford [28]	America	120	0.0700	2012	Mean, 42	63.0	ISS	44 **^a^**	7	18	1	94	0.910	88.00	84.00	8
Aydin [29]	Turkey	550	0.2160	2008	>16	78.0	ISS	21	106	92	13	339	0.907	89.10	78.70	10
							NISS	25	102	76	17	431	0.914	85.70	82.40	
Turina [24]	Croatia	43 **^b^**	0.2300	2001	Mean, 30	93.0	ISS	20	10	17	0	16	0.750	100.00	49.00	8
		41 **^c^**	0.3900	2001	Mean, 38	90.2	ISS	24	16	11	0	14	0.780	100.00	56.00	
Schiff [25]	America	294	0.0340	2002	Mean, 27.6	0.0	ISS	4	7	108	3	176	0.740	70.00	62.00	9
Domingues [13]	Brazil	533	0.2310	2011	Mean, 38	80.5	ISS	44 **^a^**	97	98	26	312	0.900	79.00	76.00	10
							NISS	54 **^a^**	101	70	22	340	0.920	82.00	83.00	
Eryllmaz [30]	Turkey	87	0.1034	2009	Mean, 25	67.0	ISS	31.5	9	24	0	54	0.910	100.00	69.20	10
							NISS	31.5	9	24	0	54	0.915	100.00	69.20	
Ahun [31]	Turkey	100	0.1200	2014	Mean, 40.35	77.0	ISS	16	10	29	2	59	0.816	83.33	67.05	10

TP, true-positive; FP, false-positive; FN, false-negative; TN, true-negative; AUC, area under the receiver operator characteristic curve; Sen, sensitivity; Spe, specificity; ISS, Injury Severity Score; NISS, New Injury Severity Score; **^a^** because the study did not report the cut-off value, we set the cut-off value according to definitions used by Osler et al. [6]; **^b^** patients with war-related ballistic injuries; **^c^** patients with non-ballistic injuries.

**Table 2:** Test of the threshold effect.

Tool	Spearman Correlation Coefficient	*p*-Value
ISS	0.517	0.085
NISS	0.300	0.624

ISS, Injury Severity Score; NISS, New Injury Severity Score.

**Table 3:** Pooled estimates of the Injury Severity Score and New Injury Severity Score.

Tool	Sensitivity (95% CI)	Specificity (95% CI)	PLR (95% CI)	NLR (95% CI)	DOR (95% CI)
ISS	0.64 (0.61–0.68)	0.93 (0.93–0.94)	5.11 (3.12–8.37)	0.27 (0.19–0.40)	27.75 (9.93–77.53)
NISS	0.71 (0.66–0.75)	0.87 (0.86–0.88)	5.22 (3.84–7.08)	0.20 (0.08–0.52)	24.74 (10.19–60.07)

CI, confidence interval; PLR, positive likelihood ratio; NLR, negative likelihood ratio; DOR, diagnostic odds ratio; ISS, Injury Severity Score; NISS, New Injury Severity Score.

**Table 4:** Results of the meta-regression analysis.

Variables	Coefficient	*p*-Value	RDOR (95% CI)
Mortality	−4.271	0.4823	0.01 (0.00–16,071.86)
Cut-off value	0.022	0.6215	1.02 (0.92–1.13)
Quality	−0.191	0.6542	0.83 (0.31–2.23)
Number	0.001	0.0805	1.00 (1.00–1.00)

CI, confidence interval; RDOR, relative diagnostic odds ratio.

**Table 5:** Results of sensitivity analysis.

First Author	Sensitivity	I^2^ of Sensitivity (%)
None **^a^**	0.64	93.2
Turina [24] **^b^**	0.64	93.4
Domingues [13]	0.62	93.2
Lefering [26]	0.70	91.6
Bulut [18]	0.64	93.4
Schiff [25]	0.64	93.8

**^a^** no study was excluded; **^b^** the patients had war-related injuries.

**Table 6:** Results of the subgroup meta-analysis for the Injury Severity Score.

Subgroup	I^2^ of Sensitivity (%)	I^2^ of Specificity (%)
All	0.64 (93.2)	0.93 (99.3)
Mortality < 0.1	0.58 (90.0)	0.96 (99.6)
Mortality ≥ 0.1	0.69 (94.5)	0.82 (93.7)
Sample size < 100	1.00 (0.0)	0.62 (56.8)
Sample size ≥ 100	0.63 (93.8)	0.94 (99.5)
Cut-off value < 44	0.69 (93.1)	0.83 (97.0)
Cut-off value ≥ 44	0.59 (94.2)	0.98 (99.6)
Developed country	0.47 (71.6)	0.83 (98.0)
Developing country	0.70 (93.3)	0.95 (99.4)
